# Synthetic graft for medial patellofemoral ligament reconstruction: a systematic review

**DOI:** 10.1186/s10195-022-00660-9

**Published:** 2022-08-22

**Authors:** Filippo Migliorini, Jörg Eschweiler, Filippo Spiezia, Matthias Knobe, Frank Hildebrand, Nicola Maffulli

**Affiliations:** 1 Department of Orthopaedic, Trauma, and Reconstructive Surgery, RWTH University Hospital, Pauwelsstraße 30, 52074 Aachen, Germany; 2 Department of Orthopaedic and Trauma Surgery, San Carlo Hospital, Potenza, Italy; 3 Department of Orthopaedic and Trauma Surgery, Cantonal Hospital, 6000 Lucerne, Switzerland; 4 Department of Medicine, Surgery and Dentistry, University of Salerno, Via S. Allende, 84081 Baronissi, SA Italy; 5 School of Pharmacy and Bioengineering, Keele University School of Medicine, Thornburrow Drive, Stoke on Trent, England; 6 Barts and the London School of Medicine and Dentistry, Centre for Sports and Exercise Medicine, Queen Mary University of London, Mile End Hospital, 275 Bancroft Road, London, E1 4DG England

**Keywords:** MPFL, Patellofemoral instability, Synthetic graft

## Abstract

**Background:**

This systematic review investigates the role of synthetic graft for primary medial patellofemoral ligament (MPFL) reconstruction in patients with recurrent patellofemoral instability, focusing on clinical scores and the rate of complications.

**Methods:**

This systematic review was conducted according to the PRISMA statement. The main online databases were accessed in January 2022 without time constraints. All clinical studies investigating the use of synthetic grafts for MPFL reconstruction were accessed. Revision settings were not considered. Only articles reporting data on patients with recurrent patellofemoral instability were eligible. Studies regarding congenital or acute patellofemoral dislocation were excluded. Only studies performing a follow-up longer than 24 months were considered.

**Results:**

Data on 199 patients [mean age 22.3 (range 19.0–28.0) years] were collected. The mean follow-up was 60.5 (39.0–142.8) months. All the scores of interest improved at last follow-up: Kujala (+ 24.8; *P* = 0.0002), Lysholm (+ 42.0; *P* = 0.02), Tegner (+ 1.2; *P* = 0.03), IKDC (+ 20.9; *P* = 0.02). Post-operatively, a positive apprehension test was detected in 6.1% (7/115) of patients, and a sensation of instability was reported by 1.5% (3/199) of patients. The rate of re-dislocations was 2.5% (5 of 199 patients), and revision procedures were performed in less than 1% (1 of 199) of patients.

**Conclusion:**

Synthetic graft may be reliable and feasible for primary MPFL reconstruction in patients with recurrent patellofemoral instability.

## Introduction

Patellofemoral instability (PFI) is common, especially in active adolescents [1–3]. The etiology of PFI is multifactorial, with several pathoanatomical risk factors predisposing to instability [4–8]. Moreover, most patients who suffer from PFI present several risk factors which synergistically predispose to instability [9–11]. Clinically, patients with PFI experience patellar subluxations and dislocations [11–13]. Lateral patellar displacement of the patella usually damages the medial patellofemoral ligament (MPFL) [14]. This ligament is the most important passive stabilizer of abnormal patellar lateralization during the first degrees of knee flexion [15, 16]. Thus, surgical MPFL reconstruction may be recommended to avoid persistent instability and further dislocations [12, 13, 17–19]. MPFL reconstruction achieves very good outcomes and patient satisfaction, along with a low rate of complications [3, 14, 20–23]. Given the greater lateralizing forces acting on the MPFL in patients with PFI [24–26], accurate reconstruction and graft selection are pivotal. While allografts and autografts are widely employed for MPFL reconstruction, the role of synthetic graft for this purpose is still unclear [18, 27–32]. Most of the literature pertaining to synthetic graft for MPFL reconstruction is based on retrospective investigations with heterogeneous criteria and results. Despite the limited evidence, results from these studies are promising. Thus, we conducted a systematic review investigating the role of synthetic graft for primary MPFL reconstruction in patients with recurrent PFI. The focus of the present study was on clinical scores and the rate of complications.

## Material and methods

### Search strategy

This systematic review was conducted according to the Preferred Reporting Items for Systematic Reviews and Meta-Analyses: the PRISMA statement [33]. A PIOT algorithm was performed preliminarily:

- P (problem): patellofemoral instability;
- I (intervention): synthetic MPFL reconstruction;
- O (outcomes): clinical scores and complications;
- T (timing): > 24 months of follow-up.

### Data source and extraction

Two authors independently (F.M. & J.E.) performed the literature search in January 2022. The following databases were accessed: PubMed, Google Scholar, Embase, and Scopus. No time constraints were set for the database search. The following keywords were used in combination:* knee*,* patella*,* patellofemoral*,* joint*,* instability*,* synthetic*,* dislocations*,* apprehension*,* subluxation*,* revision*,* failure*,* revision*,* Tegner*,* Kujala*,* Lysholm*,* score*,* graft*,* medial patellofemoral ligament*,* MPFL*,* rupture*,* tear*,* reconstruction*,* pain*,* trochlea*. The resulting articles were screened by the same two authors. The full text of the articles of interest was accessed. The bibliographies of the full-text articles were also screened. Disagreements were debated and solved by a third author (N.M.).

### Eligibility criteria

All the clinical studies investigating the role of synthetic graft for MPFL reconstruction were accessed. Given the authors’ language capabilities, articles in English, German, Italian, French and Spanish were considered. Studies of evidence of levels I–III according to the Oxford Centre of Evidence-Based Medicine [34] were eligible. Only articles reporting data on patients with recurrent PFI were eligible. Only studies performing a follow-up longer than 24 months were eligible. Only articles reporting quantitative data on the outcomes of interest were considered for inclusion. Missing data on the outcomes of interest warranted exclusion from this study. Reviews or meta-analyses, editorials, letters, expert opinions, and case reports were not considered. Articles reporting data from registries were also not eligible. Cadaveric, animal and biomechanical studies were not included; nor were articles regarding revision settings. Studies regarding congenital or acute patellofemoral dislocation were also excluded. Only studies reporting quantitative data on the outcomes of interest were included.

### Outcomes of interest

Two authors (F.M. & J.E.) independently performed data extraction. Data on study generalities (author and year, journal, study design, follow-up), baseline characteristics of the patients (number of procedures, mean age), type of graft and intervention (isolated and/or combined) were collected. The outcomes of interest were the following: the Kujala Anterior Knee Pain Scale [35], the Lysholm Knee Scoring Scale [36], the Tegner Activity Scale [37] and the International Knee Documentation Committee (IKDC) [38]. The following complications were recorded: positive apprehension test, persistent sensation of instability, and rates of revision and re-dislocation. Persistent instability was defined as recurrence and/or a subjective sensation of subluxation or instability [39, 40].

### Methodological quality assessment

To evaluate the methodological quality assessment, the Coleman Methodology Score (CMS) [41] was applied. An independent author (A.P.) performed the scoring. Part A of the CMS analyses the study size, follow-up, surgical approach, type of analysis, description of diagnosis, surgical technique and postoperative rehabilitation. Part B focuses on the outcome criteria along with related assessing procedures and the description of the subject selection process. The CMS for the quality of the study was calculated. The CMS can range from 0 (poor) to 100 (excellent), with a score of > 60 considered satisfactory.

### Statistical analysis

The statistical analyses were performed by the main author (F.M.). IBM SPSS software (version 25) was used. Continuous variables were analysed through the mean difference (MD), while the complication rate was analysed through the odds ratio (OR) effect measure. Confidence intervals (CIs) were set at 95% in all comparisons. The* t*-test was used to assess significance for continuous variables, and the $\chi$^2^ test was used for dichotomous ones. Values of *P* < 0.05 were considered statistically significant.

## Results

### Search results

**Fig. 1:**
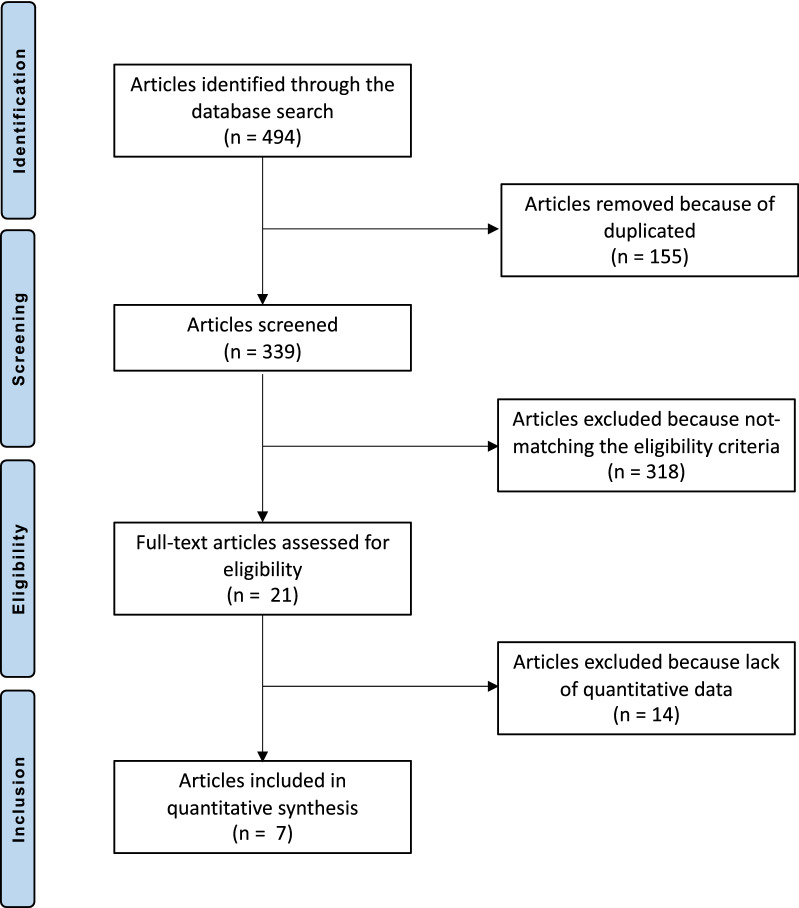
Flow chart of the literature search

The literature search resulted in 494 articles. Of these, 155 were excluded because they were duplicates. A further 332 articles were excluded because they did not match the topic (*N* = 184), they were not clinical studies or had a poor level of evidence (*N* = 92), there were language limitations (*N* = 9), they considered the treatment of acute/congenital/habitual dislocations and/or revision settings (*N* = 19), they had a short follow-up (*N* = 7), there was a lack of quantitative data on the outcomes of interest (*N* = 14), or they had a high risk of bias (e.g. they had uncertain results or a population that was too small; *N* = 7). This left seven investigations for inclusion: three prospective and four retrospective clinical studies. The flow chart of the literature search results is shown in Fig. 1.

### Methodological quality assessment

The CMS highlighted several strengths and limitations of the articles included in this study. The retrospective design of most studies represents the most important limitation. The surgical approach, diagnosis and rehabilitation were often well described, representing important strengths of this study. Criteria selection, outcome measures and related timing of assessment were adequately described. General health measures were rarely reported, while the procedures used to assess outcomes were often biased. The CMS of this study was 71 points, attesting that it provides a good methodological quality assessment. The results for the CMS are shown in Table 1.

**Table 1:** Methodological quality assessment

Endpoints		Mean	SD
Part A: Only one score to be given for each of the seven sections			
1. Study size		4.0	–
2. Mean follow-up		7.86	1.5
3. Surgical approach		9.50	1.2
4. Type of study		4.29	5.3
5. Description of diagnosis		5.00	–
6. Description of surgical technique		10.00	–
7. Description of postoperative rehabilitation		5.00	–
Part B: Scores may be given for each option in each of the three sections if applicable			
1. Outcome criteria	1.1 Outcome measures are clearly defined	2.83	1.6
	1.2 Timing of outcome assessment is clearly stated	2.33	0.5
	1.3 Use of outcome criteria that have been reported to be reliable	2.50	1.0
	1.4 General health measure is included	–	–
2. Procedure for assessing outcomes	2.1 Participants are recruited	4.17	0.4
	2.2 Investigator is independent of surgeon	1.67	1.5
	2.3 Written assessment	3.00	–
	2.4 Completion of assessment with minimal assistance	–	–
3. Description of the subject selection process	3.1 Selection criteria are reported and unbiased	3.67	0.91
	3.2 Reported recruitment rate > 80%	3.99	0.9
	3.3 Reported recruitment rate < 80%	1.01	0.3

### Patient demographics

Data from 199 patients were available. The mean age of the patients was 22.3 (19.0 to 28.0) years. The mean follow-up was 60.5 (39.0 to 142.8) months. Four studies reported a double-bundle reconstruction, while three reported a single-bundle reconstruction. The generalities and patient baselines of the included studies are shown in Table 2.

**Table 2:** Generalities and patient baselines of the included studies

Author, year	Journal	Study design	Follow-up (months)	Knees (*n*)	Mean age (years)	Type of ligament	Graft bundle
Berruto et al. 2014 [42]	*Knee Surg Sports Traumatol Arthrosc*	Prospective	40.6	18	19.0	6 mm, LARS (Orthomedic Ltd., Dollard-des-Ormeaux, Canada)	Double
Ellera Gomes et al. 1992 [43]	*Arthroscopy*	Retrospective	39	30	28.0	8 mm, polyester, Leeds-Keio (Neoligaments Ltd, Leeds, UK) Artrolig (Engimplan-Engenharia De Implante, Brazil)	Single
Khemka et al. 2016 [44]	*Knee*	Retrospective	43	31	25.0	LARS (CORIN Ltd, France), AchilloCordPLUS (Neoligaments Ltd, Leeds, UK)	Single
Lee et al. 2018 [45]	*Knee Surg Sports Traumatol Arthrosc*	Prospective	48	23	22.0	Ultra-high molecular weight polyester tape, FiberTape (Arthrex, FL, USA)	Double
Nomura et al. 2000 [46]	*Knee*	Prospective	70.8	27	21.0	15 mm, polyester, Leeds-Keio (Neoligaments Ltd, Leeds, UK)	Single
Nomura et al. 2007 [47]	*Am J Sports Med*	Retrospective	143	24	22.5	15 mm, polyester, Leeds-Keio (Neoligaments Ltd, Leeds, UK)	Single
Suganuma et al. 2016 [48]	*Arthroscopy*	Retrospective	51.6	18	20.7	20 mm, polyester, Poly-Tape PT20 (Neoligaments Ltd, Leeds, UK) 20 mm, polyester, Poly-Tape PT20 (Neoligaments Ltd, Leeds, UK)	Double
			48.0	28	20.3		

### Outcomes of interest

All the scores of interest improved at last follow-up: Kujala (+ 24.8; *P* = 0.0002), Lysholm (+ 42.0; *P* = 0.02), Tegner (+ 1.2; *P* = 0.03), and IKDC (+ 20.9; *P* = 0.02). A positive apprehension test was detected in 6.1% (7/115) of patients, while a persistent sensation of instability was present in 1.5% (3/199) of patients. The rate of re-dislocations was 2.5% (5 of 199 patients), while the rate of revision was less than 1% (1 of 199 patients). Table 3 shows the results for the scores.

**Table 3:** Results for the clinical scores

Endpoint	Pre-operative	Post-operative	MD	*P*
Kujala score	66.5 ± 7.5 (57.0–75.2)	91.3 ± 6.7 (84.0–97.7)	+ 24.8	0.0002
Lysholm score	40.5 ± 29.0 (20.0–61.0)	82.5 ± 6.4 (78.0–87.0)	+ 42.0	0.02
Tegner scale	4.0 ± 0.8 (3.0–4.6)	5.2 ± 0.7 (4.6–6.0)	+ 1.2	0.03
IKDC	60.5 ± 15.7 (42.4–69.8)	81.4 ± 10.7 (70.1–91.3)	+ 20.9	0.02

### Double-bundle vs single-bundle patellar fixation subgroups

There was similarity of the two groups at baseline concerning follow-up duration, age and number of patients (*P* > 0.1). No difference was found between single- and double-bundle reconstruction with regards to the apprehension test (OR 0.05; 95% CI: 0.0026 to 0.8261; *P* = 0.05), persistent instability (OR 0.6; 95% CI: 0.0570 to 7.1707; *P* = 0.7), re-dislocation rate (OR 0.1; 95% CI: 0.0061 to 2.0478; *P* = 0.1) and revision rate (OR 3.9; 95% CI: 0.1570 to 96.9637; *P* = 0.4). These results are shown in detail in Table 4.

**Table 4:** Results of the comparison of double-bundle vs single-bundle patellar fixation

Endpoint	Double bundle	Single bundle	95% CI	OR	*P*
Apprehension test	0/64	7/51	0.0026–0.8261	0.05	0.05
Persistent instability	1/87	2/112	0.0570–7.1707	0.6	0.7
Re-dislocation	0/87	5/112	0.0061–2.0478	0.1	0.1
Revision	1/87	0/112	0.1570–96.9637	3.9	0.4

## Discussion

According to the main findings of the present systematic review, synthetic graft can be a reliable and feasible option for primary MPFL reconstruction in patients with recurrent PFI. All the scores of interest significantly improved postoperatively, and all exceeded the relevant minimally clinically important difference (MCID) at last follow-up [37, 49, 50]. The rate of complications was similar to those reported in previous reviews concerning MPFL reconstruction with an autograft [51–54]. No difference was found between single- and double-bundle patellar fixation techniques.

McNeilan et al. [55] performed a systematic review in 2018 that analysed three studies (76 patients) in a synthetic reconstruction cohort. Similar to the main findings of the present study, synthetic grafts achieved excellent clinical outcomes, with low complication rates.

Graft choice is complex, and to date there are no agreed recommendations. Most surgeon prefer autografts. Of the several tendon autografts available, the most commonly used are gracilis and semitendinosus tendon autografts [3, 26–28, 31, 56–60] because of their intrinsic biomechanical properties [61], geometric properties [62], availability and low donor-site morbidity [63]. In the current literature, to our knowledge, there is only one study protocol for a randomized controlled trial comparing synthetic versus autologous graft for MPFL reconstruction (ISRCTN 16657952, March 2017) [64]. The ideal biomechanical properties (e.g. stiffness, viscoelasticity, tensile strength, thickness) of a graft for MPLF reconstruction remain undefined. Indeed, the tendency for lateralization of the patella is related to the presence and amount of pathoanatomical risk factors and the bone morphology. Thus, graft selection should be customized accordingly. In this context, the mechanical properties of synthetic grafts can be adapted to the surgeon’s preferences. Compared to autografts, synthetic grafts allow a shorter surgical duration and lead to less donor-site morbidity, most likely inducing less post-operative pain. Regarding the latter two issues, their prevention may favour the early phases of rehabilitation. While tendon grafts have the tendency to stretch over time, the biomechanical properties of a synthetic graft are predictable. This is important to remember during graft tensioning, since overtightening of the synthetic graft must be avoided. To avoid overtightening, Lee et al. [45] suggest tensioning the MPFL graft under direct arthroscopic vision to observe the patella position over the trochlea without the use of a thigh tourniquet. In a retrospective study, Suganuma et al. [48] investigated whether the position of the patella in the trochlea after MPFL reconstruction using a synthetic graft (Poly-Tape) affects surgical outcome. They suggest that slight undertensioning or residual lateral positioning of the patella within the trochlear groove may have a positive influence on surgical outcomes [48]. Lee et al. [45] compared synthetic versus autologous grafts for MPFL reconstruction. They reported no differences between a gracilis autograft and ultra-high-molecular-weight polyester FiberTape (Arthrex, FL, USA) in clinical outcomes and complications. Tsushima et al. [65] compared the biomechanical properties of FiberTape with a semitendinosus autograft for MPFL reconstruction. They concluded that MPFL reconstruction using FiberTape was stronger than the native MPFL, and that a semitendinosus autograft with soft-tissue anchors was weaker than FiberTape with knotless anchors. The latter achieves enough strength for MPFL reconstruction, avoiding the complications associated with graft harvesting. These considerations allow new insight and perpectives in our understanding of MPFL reconstruction.

This study does not come without limitations. The current literature lacks investigations concerning synthetic grafts for MPFL reconstruction. Consequently, the number of procedures for analysis was limited. The retrospective design and small sample sizes of most of the investigations negatively affected the reliability of the present study. This systematic review considered patients with different degrees of patellar instability. Some authors also reported data on MPFL reconstruction with combined proximal and distal alignment. However, given the lack of available data and information, it was not possible to conduct further subgroup analyses. Patients with acute patellofemoral dislocation were not included in the present study. The treatment of acute patellofemoral dislocation is controversial [17, 66–68]. Surgery is indicated as the first-line management in patients with displaced osteochondral defects or mechanical symptoms [69–71]. However, a growing tendency to treat the first patellar dislocation surgically has been evidenced [72, 73]. Given these controversies, studies which performed primary surgery in patients with acute patellofemoral dislocation were not considered. The eligibility criteria of the studies included for analysis were heterogeneous. Indeed, Nomura et al. [47] included patients with previous surgical intervention, while three of the seven included studies [42, 43, 46] did not report relevant information. Suganuma et al. [48] were the only authors who excluded patients with pathoanatomical risk factors, while there was high variability among the other included studies. Khemka et al. [44] also included patients with pathological ligamentous laxity. This heterogeneity certainly introduces an important source of bias; however, considering the lack of data in the literature, no additional subgroup analyses were possible. We must further acknowledge that two studies also combined MPFL reconstruction with tibial tuberosity transposition for different indications in a small percentage of patients. The Elmslie–Trillat procedure was performed by Berruto et al. [42] in patients with tibial tubercle-tibial groove (TT-TG) distance greater than 20 mm (5 of 18 procedures). Khemka et al. [44] performed tibial tuberosity medialization in patients with TT-TG > 15 mm (2 of 31 procedures). Moreover, they also combined every MPFL reconstruction procedure with lateral retinacular release (LRR). LRR was also performed by Nomura et al. [46, 47] in patients with severe tightness of the lateral patellar structures. Patellar and femoral graft fixation was heterogeneous among the studies, thus representing another possible source of bias. Some authors did not state whether additional surgical procedures were performed. Lastly, the dimensions and type of the synthetic ligament used was also dissimilar between the studies. Therefore, given these limitations, results from the present study must be interpreted with caution. Future studies should improve these limitations, allowing for higher-quality analyses.

## Conclusion

According to the main findings of the present systematic review, synthetic graft may be reliable and feasible for primary MPFL reconstruction in patients with recurrent patellofemoral instability. Results must be interpreted within the limitations of this study.

## Data Availability

The data underlying this article are available in the article and in its online supplementary material.

## Abbreviations

MPFL: Medial patellofemoral ligament
PFI: Patellofemoral instability
CMS: Coleman Methodology Score
MD: Mean difference
OR: Odds ratio
CI: Confidence interval
